# Facts about Cholera

**Published:** 1892-09-03

**Authors:** 


### The Doctor's Armchair.

### FACTS ABOUT CHOLERA.

If all the world consisted of well-educated physicians, cholera, as an epidemic disease would die out. In the case of cancer or of consumption cure is difficult, and prevention practically impossible. But in the case of cholera, cure, under favourable circumstances, is much less difficult, whilst prevention is comparatively easy.

At a crisis like the present, every person who has reached years of discretion should try to intelligently understand what the cause of cholera is, what the early symptoms are, what circumstances favour the spread of the disease, and what are the steps to be promptly taken by way of prevention and general management.

In the first place, let us try to definitely understand what the cause of cholera is. The cause is a poison of a very powerful kind. On this point there is no doubt whatever. The cholera bacillus is well known to science. This fact may seem to some to be a complicating mystery; but it ought to be considered rather a simplifying circumstance, as it really is. If we try to trace the origin and progress of a cholera epidemic we shall be much helped thereby to see clearly what we as individuals ought to do when the dangerous malady approaches our own homes. Let us transport ourselves in imagination to India. In that part of Asia cholera is endemic, in other words, it is always present and always more or less active. There are excellent authorities who believe that India is the natural abode of cholera, just as it is the natural abode of Bengal tigers and certain orders of snakes. The true and proper poison of Asiatic cholera is a bacillus—the "comma" bacillus—which lives and multiplies in the bowels of infected persons. When the bowels of those persons are moved the dejecta contain the bacilli, or poisonous agents. It is most important for every person to understand what the circumstances are under which the dejecta of choleraic patients communicate the disease. When once these circumstances have been clearly realised by the mind, the most efficient of all the lessons of prevention has been well learnt. Here an actual fact may help us. Shortly before the outbreak of the cholera epidemic of 1866 a small quantity of the dejecta, or "rice-water" motions, of a patient in India who was suffering from Asiatic cholera got accidentally mixed with four or five gallons of water, and the water was thereafter exposed to the heat of a tropical sun for twelve hours. Early next morning nineteen persons, evidently not noticing anything peculiar about either the appearance or the taste of the water, each drank a small quantity—not, probably, more than an ounce—about two tablespoonfuls. None of them drank oftener than once, and yet of the nineteen no fewer than five were seized with cholera within thirty-six hours.

It is manifest then that the minutest possible quantity of dejecta from the bowels of a choleraic patient, even if mixed with thousands of times its own weight of water, is a poison of the swiftest and most deadly character. To prevent the minutest trace of such a poison from getting into water pipes, cisterns, or drinking water in any form whatever, is the very head and chief of all preventive measures. To accomplish this end there is one, and only one, method which can be trusted to be invariably effectual. The patient must pass all his motions into a strong disinfecting fluid. There are dozens of such fluids available at very moderate prices, and we need not here mention any one in particular.

The next fact of importance to remember is that the dried dejecta of cholera patients are as poisonous as the moist. If the greatest possible care be not taken the patient's clothing, the bedding, and other articles in the sick-room will inevitably be soiled. If the soiled portions of bedding or clothing be allowed to dry, no matter how small those portions may be, every one of them will yield a fine powder which contains cholera bacilli, and is a deadly poison. All soiled articles must therefore be disinfected immediately. No such thing as the drying of soiled portions of clothing or bedding should be permitted even for an instant. It is not necessary that the dried dejecta be moistened with water, or even swallowed, for their poisonous effects to be produced. The mere smelling of dried choleraic dejecta is believed to be quite sufficient to bring on a dangerous, or even a fatal, attack of cholera in a susceptible person.

A third point to be borne in mind is that if the poisonous dejecta be thrown on the ground, or even buried, their poisonous character is not altered. The "comma" bacillus, which, as we have said, is the active agent in the production of cholera, seems to live and thrive exceedingly well in almost any kind of soil or common earth. The soil of a garden or field impregnated by even a small portion of cholera dejecta seems to become poisoned forthwith. So far as can be made out the deadly bacilli multiply in the ground, and years after favouring conditions may stimulate them to unusual activity, causing them to poison the superincumbent atmosphere; or they may be washed by falling showers into neighbouring wells or running streams, and there do their deadly work as efficiently as if newly separated from their abode in the bowel of a choleraic patient. All this sounds painfully alarming. But it is in reality much less alarming than it sounds. The thing to be done is to take care to destroy all the bacilli in the dejecta the moment they are passed from the bowels. If this be done, the long train of evils we have here depicted will be anticipated, and entirely prevented. The one lesson to be learnt and practised is that all cholera patients must use separate closets, or commodes, and that these must be abundantly supplied with disinfecting fluid of more than adequate strength.

This question of separate closets is of prime importance, and it leads to the mention of the necessity for the separation and isolation of cholera patients. If cholera break out in any house, one large airy room should be set aside as a hospital ward. It should be stripped of all superfluous furniture, as in the case of scarlet fever, and used for no other purpose but that of a hospital ward. In the matter of prevention and disinfection practical persons can be their own best helpers. They should at once set about learning the elements of sanitation, but above all what they have learnt they must resolutely put into practice daily and hourly until the shadow of the dark cloud of cholera has entirely disappeared from the sky.

